# Supplementary material for: Shared genetic aetiology between cognitive functions and physical and mental health in UK Biobank (N=112 151) and 24 GWAS consortia
Source: Mol Psychiatry. 2016 Jan 26;21(11):1624–32. doi: 10.1038/mp.2015.225 (PMC5078856; doi:10.1038/mp.2015.225)
Supplement: Supplementary Information [file mp2015225x1.doc]

**Supplementary Materials for:**

Hagenaars et al. Shared genetic aetiology between cognitive functions and physical and mental health in UK Biobank (N = 112 151) and 24 GWAS consortia

**Contents**

**Page 2:** Supplementary Table 1, on associations between cognitive and health variables.

**Page 3:** References for Supplementary Table 1.

**Page 5:** Descriptions of cognitive phenotypes.

**Page 6:** Supplementary Figure 1, on age associations of cognitive phenotypes in UK Biobank.

**Page 7:** Genotyping and quality control.

**Page 8:** Genome-wide association analyses (GWAS) in the UK Biobank sample.

**Page 9:** Sources of genetic results from genome-wide association studies (GWAS) consortia.

**Page 11:** Supplementary Table 2, on details of the sources of genetic results from genome-wide association studies (GWAS) consortia.

**Page 13:** LD regression genetic correlation procedure.

**Page 14:** Polygenic profiling procedure.

**Page 15:** Sensitivity analyses

**Page 16:** Supplementary Table 3, on the number of SNPs included at each threshold for the polygenic profile scores.

**Page 19:** Supplementary Tables 4a-d, on the full analyses between UK Biobank cognitive phenotypes and polygenic profile scores for 24 health-related variables including all SNP thresholds for polygenic profile score creation.

**Page 31:** Supplementary Table 5, on results of multivariate polygenic risk scores from the GWAS consortia in predicting cognitive test scores in UK Biobank subjects.

**Supplementary Table 1**

For each health-related phenotype for which we use genome-wide association study summary data, here are some key example references showing its relation to prior cognitive function (left-hand column) and its possible effects on later cognitive function (right-hand column). (-) indicates a negative association with cognitive function, while (+) indicates a positive association with cognitive function.

| Link to prior cognitive function | Phenotype | Effect on cognitive function |
| --- | --- | --- |
|  | **Vascular-metabolic diseases** |  |
| Lawlor et al. (2008). (-) | Coronary Artery Disease | Kovacic et al. (2012) (-)  Eggermont et al (2012) (-) |
| Lawlor et al. (2008) (-)  Rajan et al. (2014) (-) | Stroke: Ischaemic | Brainin et al. (2015) (-) |
| Lawlor et al. (2008) (-)  Rajan et al. (2014) (-) | Stroke: Cardioembolic | Brainin et al. (2015) (-) |
| Lawlor et al. (2008) (-)  Rajan et al. (2014) (-) | Stroke: Large Vessel Disease | Brainin et al. (2015) (-) |
| Lawlor et al. (2008) (-)  Rajan et al. (2014) (-) | Stroke: Small Vessel Disease | Brainin et al. (2015) (-) |
| Mõttus et al. (2013) (-) | Type 2 Diabetes | Rawlings et al. (2014) (-) |
|  | **Neuropsychiatric disorders** |  |
| N/A | ADHD | Frazier et al. (2004) (-) |
| Whalley et al. (2000) (-) | Alzheimer's Disease | N/A |
| N/A | Autism | Fombonne et al. (2005) (-) |
| Gale et al. (2013) (+) | Bipolar Disorder | Gildengers et al. (2009) (-) |
| Gale et al. (2008) (-) | Major Depressive Disorder | Wilson et al. (2014) (-) |
| Dickson et al. (2012) (-) | Schizophrenia | Hedman et al. (2013) (-) |
|  | **Brain measures** |  |
| Aribisala et al. (2014) (+) | Hippocampal Volume | van Petten et al. (2004) |
| Royle et al. (2013) (+) | Intracranial Volume | Pietschnig et al. (2014) (+) |
| N/A | Infant Head Circumference | Gale et al. (2003) (+) |
|  | **Physical and physiological measures** |  |
| Starr et al. (2004) (-) | Blood Pressure: Diastolic | Taylor et al. (2013) (+/-)  Novak & Hajjar (2010) (+/-) |
| Starr et al. (2004) (-) | Blood Pressure: Systolic | Gottesman et al. (2014) (-)  Novak & Hajjar (2010) (+/-) |
| Belsky et al. (2013) (-) | BMI | Dahl et al. (2010) (-) |
| No reference found. | Height | Russ et al. (2014) (+) |
| Calvin et al. (2011) (+) | Longevity | N/A |
| Richards et al. (2005) (+) | Forced Expiratory Volume in 1s | Emery et al. (2012) (+) |
|  | **Life-course Cognitive traits and proxies** |  |
| N/A | Childhood cognitive ability | Deary et al. (2013) (+) |
| Strenze (2007) (+) | College degree | Clouston et al. (2012) (+) |
| Strenze (2007) (+) | Years of Education | Banks & Mazzonna (2012) (+) |

**References for Supplementary Table 1**

Aribisala BS, Royle NA, Muñoz Maniega S, et al. Quantitative multi-modal MRI of the hippocampus and cognitive ability in community-dwelling older subjects. Cortex 2014; 53: 34-44.

Banks J, Mazzonna F. The effect of education on old age cognitive abilities: Evidence from a regression discontinuity design. Econ J 2012; 122: 418-448.

Belsky DW, Caspi A, Goldman-Mellor S, et al. Is obesity associated with a decline in intelligence quotient during the first half of the life course? Am J Epidemiol 2013; 178: 1461-1468.

Brainin M, Tuomilehto J, Heiss WD, et al. Post-stroke cognitive decline: An update and perspectives for clinical research. Eur J Neurol 2015; 22: 229-e16.

Calvin CM, Deary IJ, Fenton C, et al. Intelligence in youth and all-cause-mortality: systematic review with meta-analysis. Int J Epidemiol 2011; 40: 626-644.

Clouston SAP, Kuh D, Herd P, et al. Benefits of educational attainment on adult fluid cognition: International evidence from three birth cohorts. Int J Epidemiol 2012; 41: 1729-1736.

Dahl A, Hassing LB, Fransson E, et al. Being overweight in midlife is associated with lower cognitive ability and steeper cognitive decline in late life. J Gerontol A Biol Sci Med Sci. 2010; 65: 57-62.

Deary IJ, Pattie A, Starr JM. The stability of intelligence from age 11 to age 90 years. Psych Sci 2013; 24: 2361-2368.

Emery CF, Finkel D, Pedersen NL. Pulmonary function as a cause of cognitive aging. Psychol Sci 2012; 23: 1024-1032.

Fombonne E. The changing epidemiology of autism. J Appl Res Intellect 2005; 18: 281-294.

Frazier TW, Demaree HA, Youngstrom EA. Meta-analysis of intellectual and neuropsychological test performance in attention-deficit/hyperactivity disorder. Neuropsychology 2004; 18: 543-555.

Gale CR, Batty GD, McIntosh AM, et al. Is bipolar disorder more common in highly intelligent people? A cohort study of a million men. Mol Psychiatr 2013; 18: 190-194.

Gale CR, Deary IJ, Boyle SH, et al. Cognitive ability in early adulthood and risk of 5 specific psychiatric disorders in middle age: the Vietnam experience study. Arch Gen Psychiatry 2008; 65: 1410-1418.

Gale CR, Walton S, Martyn CN. Foetal and postnatal head growth and risk of cognitive decline in old age. Brain. 2003; 126: 2273-2278.

Gildengers AG, Mulsant BH, Begley A, et al. The longitudinal course of cognition in older adults with bipolar disorder. Bipolar Disord. 2009; 11: 744-752.

Gottesman RF, Schneider AL, Albert M, et al. Midlife hypertension and 20-year cognitive change: the atherosclerosis risk in communities neurocognitive study. JAMA Neurol 2014; 71: 1218-1227.

Dickson H, Laurens KR, Cullen AE, Hodgins, S. Meta-analyses of cognitive and motor function in youth aged 16 years and younger who subsequently develop schizophrenia. Psychological Medicine 2012; 42: 743-755.

Hedman AM, van Haren NE, van Baal CG, et al. IQ change over time in schizophrenia and healthy individuals: A meta-analysis. Schizophr Res 2013; 146: 201-208.

Kovacic, JC, Castellano JM, Fuster V. The links between complex coronary disease, cerebrovascular disease, and degenerative brain disease. Ann NY Acad Sci 2012; 1254: 99-105.

Eggermont LH, de Boer K, Muller M, et al. Cardiac disease and cognitive impairment: a systematic review. Heart 2012; 98: 1334-1340.

Lawlor DA, Batty GD, Clark H, et al. Association of childhood intelligence with risk of coronary heart disease and stroke: findings from the Aberdeen Children of the 1950s cohort study. Eur J Epidemiol 2008; 23: 695-706.

Mõttus R, Luciano M, Starr JM, Deary IJ. Diabetes and life-long cognitive ability. J Psychosom Res 2013; 75: 275-278.

Novak V, Hajjar I. The relationship between blood pressure and cognitive function. Nat Rev Cardiol 2010; 7: 686-698.

Pietschnig J, Penke L, Wicherts JM, et al. Meta-analysis of associations between human brain volume and intelligence differences: How strong are they and what do they mean? SSRN 2014; <http://papers.ssrn.com/sol3/papers.cfm?abstract_id=2512128>.

Rajan KB, Aggarwal NT, Wilson RS, et al. Association of cognitive functioning, incident stroke, and mortality in older adults. Stroke 2014; 45: 2563-2567.

Rawlings AM, Sharrett AR, Schneider AL, et al. Diabetes in midlife and cognitive change over 20 years: a cohort study. Ann Intern Med 2014; 161: 785-793.

Richards M, Strachan D, Hardy R, et al. Lung function and cognitive ability in a longitudinal birth cohort study. Psychosom Med 2005; 67: 602-608.

Royle NA, Booth T, Valdes Hernández MC, et al. Estimated maximal and current brain volume predict cognitive ability in old age. Neurobiol Aging 2013; 34: 2726-2733.

Russ TC, Kivimäki M, Starr JM, et al. Height in relation to dementia death: Individual participant meta-analysis of 18 UK prospective cohort studies. Brit J Psychiatr 2014; 205: 348-354.

Starr JM, Taylor MD, Hart CL, et al. Childhood mental ability and blood pressure at midlife: linking the Scottish Mental Survey 1932 and the Midspan Studies. J Hypertens 2004; 22: 893-897.

Strenze, T. Intelligence and socioeconomic success: A meta-analytic review of longitudinal research. Intelligence 2007; 35: 401-426.

Taylor C, Tillin T, Chaturvedi N, et al. Midlife hypertensive status and cognitive function 20 years later: the Southall and Brent revisited study. J Am Geriatr Soc 2013; 61: 1489-1498.

van Petten C. Relationship between hippocampal volume and memory ability in healthy individuals across the lifespan: Review and meta-analysis. Neuropsychologia 2004; 42: 1394-1413.

Whalley LJ, Starr JM, Athawes R, et al. Childhood mental ability and dementia. Neurology 2000; 55: 1455-1459.

Wilson RS, Capuano AW, Boyle PA, et al. Clinical-pathologic study of depressive symptoms and cognitive decline in old age. Neurology 2014; 83: 702-709.

**Descriptions of Cognitive Phenotypes**

*Reaction Time Test*

Reaction Time (RT) was measured in all participants using a computer-based ‘Snap’ game, conceptually similar to some ‘Go/No-Go’ reaction time tasks. Two cards with simple symbols (e.g. a square or equals sign), were presented to participants on a computer screen. Participants were instructed to push an adjacent button box as quickly as possible, using their dominant hand, if the two cards had identical symbols. After completing four practice trials, participants completed eight experimental trials, of which four included identical pairs; these four required a button to be pressed. Each participant’s RT score was calculated as the mean time (in ms) to push the button for the four trials in which the stimuli were identical. Owing to a positively-skewed distribution, the reaction time data were log-transformed before analysis. One participant with an outlying reaction time score (1905 ms, which is over 11 standard deviations above the mean RT) had their data removed from the analysis. The Cronbach alpha coefficient, which provides an internal consistency type of reliability, for the five trials was 0.85. The UK Biobank Field IDs used in this test were 401, 402, 403, and 404. There is a linear decline in mean performance (with longer reaction times found) between age 40 and 70 years (Supplementary Figure 1, below).

*Memory (Pairs Matching) Test*

Memory was measured using a computerised ‘pairs matching’ game. There were two rounds of the game. In the first round, three pairs of cards with matching simple symbols, arranged randomly in a grid, were presented to participants on a computer screen for three seconds. The cards were then ‘turned’ face down. The participants were instructed to select, from recall and in the fewest number of attempts, the pairs of cards that had matching symbols. Pairs were identified by the participant’s touching identical cards on the screen consecutively. There was no time limit and the participants could make as many attempts as they needed to find all the pairs. The second round included six pairs of cards, shown for five seconds. Only the results from this second, more challenging round of the memory test were used in this study. The memory test score in the present study is the total number of errors made during this task until the six pairs of identical cards were touched consecutively. Twenty-nine participants who made more than 30 errors on the task had their number of errors set to 30 (this was not performed for the GWAS analysis, therefore this does not apply to the LD regression analysis). The UK Biobank field ID for the variable used here (number of errors in the second round) was 399. There is a linear decline in mean performance (more errors) between age 40 and 70 years (Supplementary Figure 1, below).

*Verbal-numerical Reasoning Test*

This test is named ‘fluid intelligence’ in UK Biobank. This verbal-numerical reasoning test comprised thirteen questions presented serially on a computer screen. There were six verbal items. There were seven numerical items, involving sequence recognition and arithmetic. Participants were required to answer all the items within two minutes. All were multiple-choice. An example verbal item is: “Age is to years as height is to?” (answer options were, “Long/Deep/Top/Metres/Tall/Do not know/Prefer not to answer”). An example numerical item is: “150…137…125…114…104… what comes next?” (answer options were, “96/95/94/93/92/Do not know/Prefer not to answer”). The total score out of thirteen was recorded and used for the present study. The Cronbach alpha coefficient for the thirteen items was 0.62. The UK Biobank Field IDs (variable names) used in this test, with each one corresponding to each of the 13 items, were 4935, 4946, 4957, 4968, 4979, 4990, 5001, 5012, 5556, 5699, 5779, 5790, and 5866. The scores on this test show stable mean values between age 40 and 60 years, and linear decline in mean scores between age 60 and 70 years (Supplementary Figure 1, below). Therefore, this test does not, across the whole age range, show the characteristic age-related decline expected from ‘fluid’ cognitive functions—the name the test has in UK Biobank—and so we refer to the test by its content, which is verbal-numerical reasoning. The cross-sectional age-related pattern of this test is similar to that shown by vocabulary tests, which are used to assess crystallised cognitive ability (Salthouse TA, Localizing age-related individual differences in a hierarchical structure. Intelligence; 32, 541-561, Figure 3).

*Educational Attainment*

As part of the sociodemographic questionnaire in the study, participants were asked, “Which of the following qualifications do you have? (You can select more than one)”. Possible answers were: “College or University Degree/A levels or AS levels or equivalent/O levels or GCSE or equivalent/CSEs or equivalent/NVQ or HND or HNC or equivalent/Other professional qualifications e.g. nursing, teaching/None of the above/Prefer not to answer”. For the present study, a binary education variable was created to indicate whether or not a participant had a college or university-level degree. This educational attainment variable was used in this study as what has been named and validated as a ‘proxy phenotype’ for cognitive function (Rietveld CA, Esko T, Davies G, et al. Common genetic variants associated with cognitive performance identified using the proxy-phenotype method. Proc Natl Acad Sci 2014; 111: 13790-13794).

**Supplementary Figure 1**

Age trends in standardized mean scores for the three cognitive abilities (Memory, Reaction Time, and Verbal-Numerical Reasoning) in the full sample (left) and the participants with genotyping data available (right). Error bars represent +/- one standard error of the mean. Valid sample sizes are shown for each individual test. Memory and Reaction Time tests had scores reversed so that lower scores indicate poorer performance for all three tests. For the ‘Full Sample’ figure, six participants aged under 40 years and seven participants aged over 70 years were removed. For the ‘Genotyped Participants Only’ figure, one participant aged over 70 years was removed.

.

**Genotyping and Quality Control**

Genotyping was performed on 33 batches of ~4700 samples by Affymetrix, who also performed initial quality control of the genotyping data. Further details are available of the sample processing specific to the UK Biobank project (<http://biobank.ctsu.ox.ac.uk/crystal/refer.cgi?id=155583>) and the Axiom array (<http://media.affymetrix.com/support/downloads/manuals/axiom_2_assay_auto_workflow_user_guide.pdf>). Prior to the release of the UK Biobank genetic data a stringent QC protocol was applied, and performed at the Wellcome Trust Centre for Human Genetics (WTCHG); details of this process can be found at the following URL (<http://biobank.ctsu.ox.ac.uk/crystal/refer.cgi?id=155580>).

Prior to the analyses described in the main report, further quality control measures were applied by the present authors. Individuals were removed based on missingness, relatedness (KING estimated kinship co-efficient > 0.0442), gender mismatch, non-British ancestry (principal component analysis identified probable Caucasians within those individuals that were self-identified as British), and QC failure in the UK Bileve study. A sample of 112 151 individuals remained for further analyses. A minor allele frequency of maf < 1% filter was applied and only autosomal variants were used in this study (N = 705 516).

**Genome-wide association analyses (GWAS) in the UK Biobank sample**

An imputed dataset, including >70 million variants, was made available in which the UK Biobank interim release was imputed to a reference set which combined the UK10K haplotype and 1000 Genomes Phase 3 reference panels. Further details can be found at the following URL: <http://biobank.ctsu.ox.ac.uk/crystal/refer.cgi?id=157020>. Genome-wide association analyses were performed on the imputed dataset using SNPTest v2.5.1 (Marchini J, Howie B, Myers S, et al. A new multipoint method for genome-wide association studies via imputation of genotypes. Nat Genet 2007; 39: 906-913; SNPTEST v.2.5.1 can be found at the following URL: [https://mathgen.stats.ox.ac.uk/genetics_software/snptest/snptest.html#introduction](https://mathgen.stats.ox.ac.uk/genetics_software/snptest/snptest.html#introduction)). An additive model was specified using the "frequentist 1" option. To account for genotype uncertainty we analysed the expected genotyped counts (dosages). Adjustments for age, sex, genotyping batch, genotyping array, assessment centre and 10 principal components were included. Prior to use in LD regression analyses the association results were filtered based on minor allele frequency (<0.1%) and imputation quality (<0.1).

**Sources of genetic results from genome-wide association consortia**

**CARDIoGRAM**

Coronary artery disease data have been contributed by CARDIoGRAMplusC4D investigators.

**CHARGE-Aging and Longevity**

Longevity data have been provided by the CHARGE-Aging and Longevity consortium. Longevity was defined as reaching age 90 years or older. Genotyped participants who died between the ages of 55 and 80 years were used as the control group. There were 6036 participants who achieved longevity and 3757 participants in the control group across participating studies in the discovery meta-analysis.

Broer L, Buchman AS, Deelen J, Evans DS, Faul JD, Lunetta KL, Sebastiani P, Smith JA, Smith AV, Tanaka T, Yu L, Arnold AM, Aspelund T, Benjamin EJ, De Jager PL, Eirkisdottir G, Evans DA, Garcia ME, Hofman A, Kaplan RC, Kardia SL, Kiel DP, Oostra BA, Orwoll ES, Parimi N, Psaty BM, Rivadeneira F, Rotter JI, Seshadri S, Singleton A, Tiemeier H, Uitterlinden AG, Zhao W, Bandinelli S, Bennett DA, Ferrucci L, Gudnason V, Harris TB, Karasik D, Launer LJ, Perls TT, Slagboom PE, Tranah GJ, Weir DR, Newman AB, van Duijn CM and Murabito JM. **GWAS of Longevity in CHARGE Consortium Confirms APOE and FOXO3 Candidacy**. *J Gerontol A Biol Sci Med Sci*. 2015;70:110-8.

*Acknowledgments*

The CHARGE Aging and Longevity working group analysis of the longevity phenotype was funded through the individual contributing studies. The working group thanks all study participants and study staff**.**

**DIAGRAM**

Type 2 diabetes data were obtained from the DIAGRAM consortium.

**International Consortium of Blood Pressure (ICBP)**

Blood pressure data were provided by ICBP.

**Early Growth Genetics Consortium (EGG)**

Head circumference data has been contributed by the EGG Consortium.

**ENIGMA**

Brain imaging data were obtained from the ENIGMA consortium.

**GIANT**

Height and BMI data were obtained from the GIANT consortium.

**International Genomics of Alzheimer’s Project (IGAP)**

Alzheimer’s disease data were obtained from (IGAP)

*Material and methods*

International Genomics of Alzheimer's Project (IGAP) is a large two-stage study based upon genome-wide association studies (GWAS) on individuals of European ancestry. In stage 1, IGAP used genotyped and imputed data on 7 055 881 single nucleotide polymorphisms (SNPs) to meta-analyse four previously-published GWAS datasets consisting of 17 008 Alzheimer's disease cases and 37 154 controls (The European Alzheimer's disease Initiative – EADI the Alzheimer Disease Genetics Consortium – ADGC The Cohorts for Heart and Aging Research in Genomic Epidemiology consortium – CHARGE The Genetic and Environmental Risk in AD consortium – GERAD). In stage 2, 11 632 SNPs were genotyped and tested for association in an independent set of 8572 Alzheimer's disease cases and 11 312 controls. Finally, a meta-analysis was performed combining results from stages 1 & 2.

*Acknowledgments*

We thank the International Genomics of Alzheimer's Project (IGAP) for providing summary results data for these analyses. The investigators within IGAP contributed to the design and implementation of IGAP and/or provided data but did not participate in analysis or writing of this report. IGAP was made possible by the generous participation of the control subjects, the patients, and their families. The i–Select chips was funded by the French National Foundation on Alzheimer's disease and related disorders. EADI was supported by the LABEX (laboratory of excellence program investment for the future) DISTALZ grant, Inserm, Institut Pasteur de Lille, Université de Lille 2 and the Lille University Hospital. GERAD was supported by the Medical Research Council (Grant n° 503480), Alzheimer's Research UK (Grant n° 503176), the Wellcome Trust (Grant n° 082604/2/07/Z) and German Federal Ministry of Education and Research (BMBF): Competence Network Dementia (CND) grant n° 01GI0102, 01GI0711, 01GI0420. CHARGE was partly supported by the NIH/NIA grant R01 AG033193 and the NIA AG081220 and AGES contract N01–AG–12100, the NHLBI grant R01 HL105756, the Icelandic Heart Association, and the Erasmus Medical Center and Erasmus University. ADGC was supported by the NIH/NIA grants: U01 AG032984, U24 AG021886, U01 AG016976, and the Alzheimer's Association grant ADGC–10–196728.

**METASTROKE**

Ischaemic stroke data were obtained from the METASTROKE consortium. The METASTROKE consortium is supported by NINDS (NS017950). We thank all study participants, volunteers, and study personnel that made this consortium possible. The METASTROKE study consists of combined data from 15 GWAS of IS (12 389 cases vs 62 004 controls). We used TOAST criteria17 to classify IS as large artery stroke (LAS) (2167 cases/49 159 controls from 11 studies), cardioembolic stroke (CE) (2365 cases/ 56,140 controls from 13 studies), and small vessel disease (SVD) (1894 cases/51 976 controls from 12 studies). METASTROKE studies consisted of independently performed genome-wide single nucleotide polymorphism (SNP) genotyping using standard technologies and imputation to HapMap release 21 or 22 CEU phased genotype18 or 1000 Genome

reference panels. Investigators contributed summary statistical data from association analyses using frequentist additive models for metaanalysis after application of appropriate quality control measures. Polygenic scores reveal combined effects of multiple nonsignificant variants derived from a derivation sample and tested in an

independent replication sample. We derived polygenic scores for multiple p value cutoffs (0.5, 0.25, 0.1, 0.05, 0.01, 0.001, and 0.0001) in derivation samples.

**Psychiatric Genetics Consortium**

Schizophrenia, bipolar disorder, major depressive disorder, ADHD and autism data were obtained from the Psychiatric Genetics Consortium.

**Social Science Genetic Association Consortium**

Years of education, college degree and childhood cognitive ability data were obtained from the Social Science Genetic Association Consortium.

**SpiroMeta/CHARGE-Pulmonary**

Lung function data were obtained from the SpiroMeta and CHARGE-Pulmonary consortia.

**Supplementary Table 2**

Sources of genetic results from genome-wide association consortia.

| **Phenotype** | **Consortium** | **URL** | **Reference** | **No. of individuals in GWAS** |
| --- | --- | --- | --- | --- |
| Coronary Artery Disease | CARDIoGRAM | http://www.cardiogramplusc4d.org/downloads/ | Schunkert et al. Nat Genet 2011; 43: 333-338. PMID: 21378990 | 22 233 cases  64 762 controls |
| Stroke:Ischaemic | METASTROKE | http://www.strokegenetics.com/members-area/meta-stroke | Traylor et al. Lancet Neurol 2012; 11: 951-962. PMID: 23041239 | 12 389 cases  62,004 controls |
| Stroke: Cardioembolic | METASTROKE | http://www.strokegenetics.com/members-area/meta-stroke | Traylor et al. Lancet Neurol 2012; 11: 951-962. PMID: 23041239 | 2365 cases  62 004 controls |
| Stroke: Large-vessel disease | METASTROKE | http://www.strokegenetics.com/members-area/meta-stroke | Traylor et al. Lancet Neurol 2012; 11: 951-962. PMID: 23041239 | 2167 cases  62 004 controls |
| Stroke: Small-vessel disease | METASTROKE | http://www.strokegenetics.com/members-area/meta-stroke | Traylor et al. Lancet Neurol 2012; 11: 951-962. PMID: 23041239 | 1894 cases  62 004 controls |
| Type 2 diabetes | DIAGRAM | http://diagram-consortium.org/downloads.html | Morris et al. Nat Genet 2012; 44: 981-990. PMID: 22885922 | 12 171 cases  56 862 controls |
| ADHD | Psychiatric Genetics Consortium (PGC) | https://www.med.unc.edu/pgc/downloads | Cross-Disorder Group of the Psychiatric Genomics Consortium. Lancet 2013; 381: 1371-1379. PMID: 23453885 | 1947 trio cases 1947 trio pseudocontrols, 840 cases  688 controls |
| Alzheimer's disease | International Genomics of Alzheimer’s Project (IGAP) | http://www.pasteur-lille.fr/en/recherche/u744/igap/igap_download.php | Lambert et al. Nat Genet 2013; 45: 1452-1458. PMID: 24162737 | 17 008 cases  37 154 controls |
| Autism | Psychiatric Genetics Consortium (PGC) | https://www.med.unc.edu/pgc/downloads | Cross-Disorder Group of the Psychiatric Genomics Consortium. Nat Genet 2013: 45; 984-994. PMID: 23933821 | 3303 cases  3428 controls |
| Bipolar disorder | Psychiatric Genetics Consortium (PGC) | https://www.med.unc.edu/pgc/downloads | Psychiatric GWAS Consortium Bipolar Disorder Working Group. Nat Genet 2011; 43: 977-983. PMID: 21926972 | 7481 cases  9250 controls |
| Major depressive disorder | Psychiatric Genetics Consortium (PGC) | https://www.med.unc.edu/pgc/downloads | Major Depressive Disorder Working Group of the Psychiatric GWAS Consortium. Mol Psychiatr 2013; 18: 497-511. PMID: 22472876 | 9240 cases  9519 controls |
| Schizophrenia | Psychiatric Genetics Consortium (PGC) | https://www.med.unc.edu/pgc/downloads | Schizophrenia Working Group of the Psychiatric Genomics Consortium. Nature 2014; 511: 421-427. PMID: 25056061 | 36 989 cases  113 075 controls |
| Hippocampal volume | ENIGMA | http://enigma.ini.usc.edu/ongoing/gwasma-of-subcorticalstructures/ | Hibar et al. Nature 2015; 520: 224-229. PMID: 25607358 | 13 171 |
| Intracranial volume | ENIGMA | http://enigma.ini.usc.edu/ongoing/gwasma-of-subcorticalstructures/ | Hibar et al. Nature 2015; 520: 224-229. PMID: 25607358 | 13 171 |
| BMI | GIANT | http://www.broadinstitute.org/collaboration/ giant/index.php/GIANT_  consortium_data_files | Locke et al. Nature 2015; 518: 197-206. PMID: 25673413 | 339 224 |
| Diastolic blood pressure | International Consortium of Blood Pressure (ICBP) |  | Ehret et al. (2011) Nature 478, 103-109. PMID: 21909115 | 69 395 |
| Height | GIANT | http://www.broadinstitute.org/collaboration/  giant/index.php/GIANT_  consortium_data_files | Wood et al. Nat Genet 2014; 11: 1173-86. PMID: 25282103 | 253 288 |
| Infant head circumference | Early Growth Genetics Consortium | http://egg-consortium.org/head-circumference.html | Taal et al. Nat Genet 2012; 15: 532-538. PMID: 22504419 | 10 768 |
| Longevity | CHARGE-Aging and Longevity |  | Broer et al. J Gerontol A Biol Sci Med Sci 2015; 70: 110-118. PMID: 25199915 | 6036 cases  3757 controls |
| Forced expiratory volume in 1 second (FEV1) | SpiroMeta/CHARGE-Pulmonary |  | Soler Artigas et al. Nature Genetics 2011; 43: 1082-1090. PMID: 21946350 | 48 201 |
| Systolic blood pressure | International Consortium of Blood Pressure (ICBP) |  | Ehret et al. Nature 2011; 478: 103-109. PMID: 21909115 | 69 395 |
| Childhood cognitive ability | Social Science Genetic Association Consortium | http://ssgac.org/Data.php | Benyamin et al. Mol Psychiatr 2014; 19: 253-258. PMID: 23358156 | 17 989 |
| College degree | Social Science Genetic Association Consortium | http://ssgac.org/Data.php | Rietveld et al. Science 2013; 314: 1467-1471. PMID: 23722424 | 95 427 |
| Years of Education | Social Science Genetic Association Consortium | http://ssgac.org/Data.php | Rietveld et al. Science 2013; 314: 1467-1471. PMID: 23722424 | 101 069 |

**LD regression genetic correlation procedure**

The patterns of LD found across the genome enable genetic correlations between traits to be derived. This is due to two reasons. Firstly, the level of association a SNP shows in a GWAS is a product of both its own contribution toward a phenotype and those that are in LD with it ( Yang J, Weedon MN, Purcell S, et al. Genomic inflation factors under polygenic inheritance. Eur J Hum Genet 2011;19: 807-812). Additionally, SNPs in regions of high LD tag a greater proportion of the genome than SNPs in regions of low LD. These two facts mean that, assuming a polygenic architecture, SNPs in regions of high LD will have greater association statistics than SNPs found in regions of low LD. The effect of this is that GWAS association test statistics can be predicted using LD (Bulik-Sullivan B, Finucane HK, Anttila V et al. An atlas of genetic correlations across human diseases and traits. bioRxiv 2015; doi: <http://dx.doi.org/10.1101/014498>). This logic can also be extended to a bivariate design, where LD can be used to predict the product of pairs of test statistics for each locus across GWAS datasets (Bulik-Sullivan B, Loh PR, Finucane H, et al. LD score regression distinguishes confounding from polygenicity in genome-wide association studies. Nat Genet 2015; 47: 291-295).

We followed the protocol of the above studies by Bulik-Sullivan et al., where data sets demonstrating a heritability Z-score (h^2^_z_ ) > 4 and a mean χ^2^ statistic of > 1.02 were included. All traits, except small vessel disease exceeded these thresholds. This threshold was implemented in order to establish that each GWAS data set had evidence of a clear polygenic signal. Where it was included in the summary statistics provided, a MAF of > 0.01 was used as a cut off. To control for imputation quality, only those SNPs found in the HapMap3 with 1000 Genomes EUR with a MAF > 0.05 were included (integrated_phase1_v3.20101123). Next, indels and structural variants were removed along with strand-ambiguous SNPs. Finally, genome-wide significant SNPs were removed, as were SNPs with very large effect sizes (χ^2^ > 80), as the presence of outliers can increase the standard error in a regression model. LD scores and weights for use with the GWAS of European ancestry were downloaded from the Broad institute (http://www.broadinstitute.org/~bulik/eur_ldscores/). An unconstrained intercept was used in the regression model as it was not possible to quantify the degree of sample overlap between the traits used here.

**Polygenic profiling procedure**

The genetic data files (.map and .ped files) supplied from Biota (the UK Biobank online repository) were unsuitable for use in the polygenic profile analyses as the .ped allele coding used a 1, 2 numeric allele encode rather than the standard ACGT encode format. In order to enable the analysis, the .ped files were recoded to the standard encode format. To achieve this, a bespoke programme was developed to create new files using a lookup-substitution method. A fast-in-memory lookup string hash table was created to hold the SNP-ID, along with the allele identifiers for the SNP. A simple loop then performed serialised lookups based on string position, to create an associated string with the correct ACGT encode. This was then appended to the six mandatory data fields extracted from initial string. In order to maximise performance and enable timely completion of the lookup-substitution, these loops were run in parallel threads in a standard multiprocessor environment.

**Sensitivity analysis**

To test whether FDR significant associations between polygenic risk for coronary artery disease, and Verbal-numerical reasoning and educational attainment were confounded by individuals diagnosed with cardiovascular disease, 2779 individuals who had had a heart attack and 2521 individuals with angina were removed from the regression analysis. Similarly 5800 individuals with diabetes (type 1 or type 2) were removed from the regression investigating an association between polygenic risk for type 2 diabetes and educational attainment. Finally, 26 912 individuals with hypertension were removed from the regression analysis investigating the association between polygenic risk for systolic blood pressure and educational attainment.

**Supplementary Table 3**

Number of SNPs included at each threshold for the polygenic profile scores.

|  |  | **Threshold** | **Number of SNPs** |
| --- | --- | --- | --- |
| **Vascular-Metabolic Diseases** | Coronary Artery Disease | 0.01 | 2633 |
|  |  | 0.05 | 9981 |
|  |  | 0.1 | 18159 |
|  |  | 0.5 | 72443 |
|  |  | 1 | 121718 |
|  | Stroke: Ischaemic | 0.01 | 3006 |
|  |  | 0.05 | 13448 |
|  |  | 0.1 | 25588 |
|  |  | 0.5 | 105309 |
|  |  | 1 | 173745 |
|  | Stroke: Cardioembolic | 0.01 | 2885 |
|  |  | 0.05 | 13295 |
|  |  | 0.1 | 25366 |
|  |  | 0.5 | 104731 |
|  |  | 1 | 172809 |
|  | Stroke: Large Vessel Disease | 0.01 | 2915 |
|  |  | 0.05 | 13354 |
|  |  | 0.1 | 25243 |
|  |  | 0.5 | 104188 |
|  |  | 1 | 172973 |
|  | Stroke: Small Vessel Disease | 0.01 | 2853 |
|  |  | 0.05 | 13116 |
|  |  | 0.1 | 25303 |
|  |  | 0.5 | 104042 |
|  |  | 1 | 172697 |
|  | Type 2 Diabetes | 0.01 | 2461 |
|  |  | 0.05 | 9969 |
|  |  | 0.1 | 18172 |
|  |  | 0.5 | 73505 |
|  |  | 1 | 124964 |
| **Neuro-Psychiatric Disorders** | ADHD | 0.01 | 1394 |
|  |  | 0.05 | 6357 |
|  |  | 0.1 | 12305 |
|  |  | 0.5 | 55047 |
|  |  | 1 | 98438 |
|  | Alzheimer's Disease | 0.01 | 5030 |
|  |  | 0.05 | 20437 |
|  |  | 0.1 | 37843 |
|  |  | 0.5 | 152714 |
|  |  | 1 | 254358 |
|  | Autism | 0.01 | 4736 |
|  |  | 0.05 | 22299 |
|  |  | 0.1 | 43182 |
|  |  | 0.5 | 192694 |
|  |  | 1 | 334262 |
|  | Bipolar Disorder | 0.01 | 2625 |
|  |  | 0.05 | 10418 |
|  |  | 0.1 | 18907 |
|  |  | 0.5 | 75289 |
|  |  | 1 | 127481 |
|  | Major Depressive Disorder | 0.01 | 1532 |
|  |  | 0.05 | 6878 |
|  |  | 0.1 | 12995 |
|  |  | 0.5 | 56175 |
|  |  | 1 | 98450 |
|  | Schizophrenia | 0.01 | 12006 |
|  |  | 0.05 | 35598 |
|  |  | 0.1 | 58837 |
|  |  | 0.5 | 194510 |
|  |  | 1 | 310629 |
| **Brain Measures** | Hippocampal Volume | 0.01 | 3954 |
|  |  | 0.05 | 17974 |
|  |  | 0.1 | 34469 |
|  |  | 0.5 | 148964 |
|  |  | 1 | 252716 |
|  | Intracranial Volume | 0.01 | 4046 |
|  |  | 0.05 | 18683 |
|  |  | 0.1 | 35510 |
|  |  | 0.5 | 150389 |
|  |  | 1 | 253355 |
|  | Infant Head Circumference | 0.01 | 2031 |
|  |  | 0.05 | 9004 |
|  |  | 0.1 | 16994 |
|  |  | 0.5 | 73859 |
|  |  | 1 | 127665 |
| **Physical and physiological measures** | Blood Pressure: Diastolic | 0.01 | 1952 |
|  |  | 0.05 | 8535 |
|  |  | 0.1 | 16298 |
|  |  | 0.5 | 74773 |
|  |  | 1 | 133754 |
|  | Blood Pressure: Systolic | 0.01 | 1961 |
|  |  | 0.05 | 8379 |
|  |  | 0.1 | 16253 |
|  |  | 0.5 | 74616 |
|  |  | 1 | 133823 |
|  | BMI | 0.01 | 2612 |
|  |  | 0.05 | 8503 |
|  |  | 0.1 | 15648 |
|  |  | 0.5 | 72010 |
|  |  | 1 | 131142 |
|  | Height | 0.01 | 11360 |
|  |  | 0.05 | 23651 |
|  |  | 0.1 | 34188 |
|  |  | 0.5 | 86984 |
|  |  | 1 | 131070 |
|  | Longevity | 0.01 | 2023 |
|  |  | 0.05 | 9292 |
|  |  | 0.1 | 17646 |
|  |  | 0.5 | 77146 |
|  |  | 1 | 133920 |
|  | Forced Expiratory Volume in 1s (FEV_1_) | 0.01 | 1963 |
|  |  | 0.05 | 8677 |
|  |  | 0.1 | 16794 |
|  |  | 0.5 | 76920 |
|  |  | 1 | 136779 |
| **Life-course Cognitive Traits and Proxies** | Childhood Cognitive Ability | 0.01 | 1187 |
|  |  | 0.05 | 4945 |
|  |  | 0.1 | 9069 |
|  |  | 0.5 | 37967 |
|  |  | 1 | 65356 |
|  | College degree | 0.01 | 2715 |
|  |  | 0.05 | 10076 |
|  |  | 0.1 | 17650 |
|  |  | 0.5 | 66808 |
|  |  | 1 | 109983 |
|  | Years of Education | 0.01 | 2702 |
|  |  | 0.05 | 9886 |
|  |  | 0.1 | 17577 |
|  |  | 0.5 | 65990 |
|  |  | 1 | 108127 |

**Supplementary Table 4a**

Associations between polygenic profiles of health related traits, and verbal-numerical reasoning controlling for age, sex, assessment centre, genotyping batch and array, and ten principal components for population structure. Statistically significant values (P<0.0188) are shown in bold. Cognitive/education phenotypes are scored such that higher scores indicate better performance.

| **Trait category** | **Trait** | **Verbal-Numerical Reasoning** | | | | |
| --- | --- | --- | --- | --- | --- | --- |
|  |  | Threshold | β | SE | R2 | P |
| **Vascular-Metabolic Diseases** | Coronary Artery Disease | 0.01 | −0.017 | 0.0052 | 0.0003 | **0.0009** |
|  |  | 0.05 | −0.012 | 0.0052 | 0.0002 | 0.0188 |
|  |  | 0.1 | −0.011 | 0.0052 | 0.0001 | 0.0393 |
|  |  | 0.5 | −0.019 | 0.0052 | 0.0004 | **0.0002** |
|  |  | 1 | −0.018 | 0.0052 | 0.0003 | **0.0005** |
|  | Stroke: Ischaemic | 0.01 | −0.010 | 0.0053 | 0.0001 | 0.0474 |
|  |  | 0.05 | −0.014 | 0.0053 | 0.0002 | **0.0068** |
|  |  | 0.1 | −0.006 | 0.0053 | 3.47×10^−5^ | 0.2604 |
|  |  | 0.5 | −0.007 | 0.0053 | 4.42×10^−5^ | 0.2041 |
|  |  | 1 | −0.003 | 0.0053 | 1.02×10^−5^ | 0.5428 |
|  | Stroke: Cardioembolic | 0.01 | 0.005 | 0.0052 | 2.45×10^−5^ | 0.3444 |
|  |  | 0.05 | 0.000 | 0.0052 | 8.40×10^−8^ | 0.9558 |
|  |  | 0.1 | −0.005 | 0.0052 | 2.06×10^−5^ | 0.3858 |
|  |  | 0.5 | −0.008 | 0.0052 | 0.0001 | 0.1092 |
|  |  | 1 | −0.009 | 0.0052 | 0.0001 | 0.0937 |
|  | Stroke: Large Vessel Disease | 0.01 | −0.013 | 0.0053 | 0.0002 | **0.0155** |
|  |  | 0.05 | −0.004 | 0.0053 | 1.28×10^−5^ | 0.4948 |
|  |  | 0.1 | −0.007 | 0.0053 | 4.24×10^−5^ | 0.2134 |
|  |  | 0.5 | −0.002 | 0.0052 | 4.11×10^−6^ | 0.6986 |
|  |  | 1 | −0.010 | 0.0052 | 0.0001 | 0.0629 |
|  | Stroke: Small Vessel Disease | 0.01 | −0.005 | 0.0052 | 2.99×10^−5^ | 0.2962 |
|  |  | 0.05 | −0.012 | 0.0053 | 0.0001 | 0.0250 |
|  |  | 0.1 | −0.007 | 0.0053 | 4.34×10^−5^ | 0.2085 |
|  |  | 0.5 | 0.001 | 0.0052 | 8.21×10^−7^ | 0.8626 |
|  |  | 1 | 0.001 | 0.0052 | 1.26×10^−6^ | 0.8300 |
|  | Type 2 Diabetes | 0.01 | −0.002 | 0.0053 | 5.84×10^−6^ | 0.6446 |
|  |  | 0.05 | −0.001 | 0.0053 | 1.63×10^−6^ | 0.8073 |
|  |  | 0.1 | −0.004 | 0.0053 | 1.60×10^−5^ | 0.4442 |
|  |  | 0.5 | −0.006 | 0.0054 | 2.98×10^−5^ | 0.2969 |
|  |  | 1 | −0.006 | 0.0054 | 3.64×10^−5^ | 0.2490 |
| **Neuro-Psychiatric Disorders** | ADHD | 0.01 | −0.002 | 0.0052 | 5.74×10^−6^ | 0.6474 |
|  |  | 0.05 | −0.001 | 0.0052 | 3.12×10^−7^ | 0.9150 |
|  |  | 0.1 | −0.004 | 0.0052 | 1.67×10^−5^ | 0.4354 |
|  |  | 0.5 | −0.007 | 0.0052 | 0.0001 | 0.1597 |
|  |  | 1 | −0.008 | 0.0052 | 0.0001 | 0.1054 |
|  | Alzheimer's Disease | 0.01 | −0.014 | 0.0052 | 0.0002 | **0.0061** |
|  |  | 0.05 | −0.023 | 0.0053 | 0.0005 | **1.27×10^−5^** |
|  |  | 0.1 | −0.023 | 0.0053 | 0.0005 | **1.34×10^−5^** |
|  |  | 0.5 | −0.021 | 0.0053 | 0.0004 | **0.0001** |
|  |  | 1 | −0.022 | 0.0053 | 0.0005 | **3.97×10^−5^** |
|  | Autism | 0.01 | 0.016 | 0.0052 | 0.0003 | **0.0023** |
|  |  | 0.05 | 0.016 | 0.0052 | 0.0002 | **0.0028** |
|  |  | 0.1 | 0.019 | 0.0052 | 0.0003 | **0.0004** |
|  |  | 0.5 | 0.022 | 0.0052 | 0.0005 | **1.92×10^−5^** |
|  |  | 1 | 0.023 | 0.0052 | 0.0005 | **1.43×10^−5^** |
|  | Bipolar Disorder | 0.01 | −0.006 | 0.0052 | 3.34×10^−5^ | 0.2695 |
|  |  | 0.05 | −0.003 | 0.0053 | 7.87×10^−6^ | 0.5920 |
|  |  | 0.1 | −0.003 | 0.0053 | 1.01×10^−5^ | 0.5433 |
|  |  | 0.5 | −0.004 | 0.0053 | 1.24×10^−5^ | 0.5006 |
|  |  | 1 | −0.004 | 0.0053 | 1.59×10^−5^ | 0.4463 |
|  | Major Depressive Disorder | 0.01 | −0.008 | 0.0053 | 0.0001 | 0.1455 |
|  |  | 0.05 | −0.008 | 0.0053 | 0.0001 | 0.1366 |
|  |  | 0.1 | −0.014 | 0.0053 | 0.0002 | **0.0071** |
|  |  | 0.5 | −0.018 | 0.0053 | 0.0003 | **0.0005** |
|  |  | 1 | −0.020 | 0.0053 | 0.0004 | **0.0002** |
|  | Schizophrenia | 0.01 | −0.055 | 0.0053 | 0.0030 | **2.04×10^−25^** |
|  |  | 0.05 | −0.062 | 0.0053 | 0.0038 | **7.73×10^−32^** |
|  |  | 0.1 | −0.062 | 0.0053 | 0.0037 | **3.22×10^−31^** |
|  |  | 0.5 | −0.062 | 0.0054 | 0.0037 | **2.59×10^−31^** |
|  |  | 1 | −0.062 | 0.0054 | 0.0036 | **1.34×10^−30^** |
| **Brain Measures** | Hippocampal Volume | 0.01 | 0.005 | 0.0052 | 2.34×10^−5^ | 0.3552 |
|  |  | 0.05 | 0.000 | 0.0052 | 1.05×10^−8^ | 0.9844 |
|  |  | 0.1 | −0.002 | 0.0052 | 4.24×10^−6^ | 0.6943 |
|  |  | 0.5 | 0.002 | 0.0052 | 2.78×10^−6^ | 0.7500 |
|  |  | 1 | −0.001 | 0.0052 | 3.26×10^−7^ | 0.9132 |
|  | Intracranial Volume | 0.01 | 0.013 | 0.0053 | 0.0002 | **0.0113** |
|  |  | 0.05 | 0.009 | 0.0053 | 0.0001 | 0.0895 |
|  |  | 0.1 | 0.013 | 0.0053 | 0.0002 | **0.0140** |
|  |  | 0.5 | 0.016 | 0.0054 | 0.0003 | **0.0021** |
|  |  | 1 | 0.016 | 0.0054 | 0.0003 | **0.0023** |
|  | Infant Head Circumference | 0.01 | 0.019 | 0.0052 | 0.0003 | **0.0004** |
|  |  | 0.05 | 0.019 | 0.0053 | 0.0003 | **0.0004** |
|  |  | 0.1 | 0.020 | 0.0053 | 0.0004 | **0.0002** |
|  |  | 0.5 | 0.024 | 0.0053 | 0.0005 | **8.53×10^−6^** |
|  |  | 1 | 0.023 | 0.0053 | 0.0005 | **1.51×10^−5^** |
| **Physical and physiological measures** | Blood Pressure: Diastolic | 0.01 | 0.001 | 0.0052 | 1.53×10^−6^ | 0.8132 |
|  |  | 0.05 | 0.004 | 0.0052 | 1.22×10^−5^ | 0.5044 |
|  |  | 0.1 | 0.000 | 0.0053 | 6.52×10^−8^ | 0.9611 |
|  |  | 0.5 | 0.003 | 0.0053 | 7.91×10^−6^ | 0.5911 |
|  |  | 1 | 0.003 | 0.0053 | 9.49×10^−6^ | 0.5563 |
|  | Blood Pressure: Systolic | 0.01 | 0.008 | 0.0052 | 0.0001 | 0.1138 |
|  |  | 0.05 | 0.008 | 0.0052 | 0.0001 | 0.1324 |
|  |  | 0.1 | 0.013 | 0.0052 | 0.0002 | **0.0116** |
|  |  | 0.5 | 0.007 | 0.0053 | 0.0001 | 0.1582 |
|  |  | 1 | 0.008 | 0.0053 | 0.0001 | 0.1211 |
|  | BMI | 0.01 | −0.022 | 0.0052 | 0.0005 | **2.92×10^−5^** |
|  |  | 0.05 | −0.024 | 0.0052 | 0.0006 | **3.44×10^−6^** |
|  |  | 0.1 | −0.024 | 0.0052 | 0.0006 | **6.49×10^−6^** |
|  |  | 0.5 | −0.027 | 0.0052 | 0.0007 | **3.34×10^−7^** |
|  |  | 1 | −0.026 | 0.0052 | 0.0007 | **8.52×10^−7^** |
|  | Height | 0.01 | 0.017 | 0.0053 | 0.0003 | **0.0011** |
|  |  | 0.05 | 0.019 | 0.0053 | 0.0004 | **0.0003** |
|  |  | 0.1 | 0.019 | 0.0054 | 0.0003 | **0.0004** |
|  |  | 0.5 | 0.021 | 0.0054 | 0.0004 | **0.0001** |
|  |  | 1 | 0.021 | 0.0054 | 0.0004 | **0.0001** |
|  | Longevity | 0.01 | 0.004 | 0.0053 | 1.87×10^−5^ | 0.4091 |
|  |  | 0.05 | 0.000 | 0.0053 | 4.62×10^−8^ | 0.9673 |
|  |  | 0.1 | 0.000 | 0.0052 | 7.30×10^−8^ | 0.9588 |
|  |  | 0.5 | 0.000 | 0.0052 | 1.31×10^−7^ | 0.9449 |
|  |  | 1 | 0.000 | 0.0052 | 1.98×10^−8^ | 0.9786 |
|  | Forced Expiratory Volume in 1s (FEV_1_) | 0.01 | 0.000 | 0.0052 | 2.08×10^−7^ | 0.9306 |
|  |  | 0.05 | 0.007 | 0.0052 | 0.0001 | 0.1604 |
|  |  | 0.1 | 0.007 | 0.0052 | 4.38×10^−5^ | 0.2064 |
|  |  | 0.5 | 0.012 | 0.0052 | 0.0001 | 0.0220 |
|  |  | 1 | 0.011 | 0.0052 | 0.0001 | 0.0319 |
| **Life-course Cognitive Traits and Proxies** | Childhood Cognitive Ability | 0.01 | 0.031 | 0.0052 | 0.0010 | **3.12×10^−9^** |
|  |  | 0.05 | 0.052 | 0.0052 | 0.0027 | **2.55×10^−23^** |
|  |  | 0.1 | 0.063 | 0.0052 | 0.0040 | **1.45×10^−33^** |
|  |  | 0.5 | 0.077 | 0.0052 | 0.0059 | **1.70×10^−48^** |
|  |  | 1 | 0.079 | 0.0052 | 0.0062 | **3.49×10^−51^** |
|  | College degree | 0.01 | 0.069 | 0.0052 | 0.0048 | **3.92×10^−40^** |
|  |  | 0.05 | 0.085 | 0.0052 | 0.0071 | **1.05×10^−58^** |
|  |  | 0.1 | 0.095 | 0.0052 | 0.0089 | **5.93×10^−73^** |
|  |  | 0.5 | 0.102 | 0.0052 | 0.0102 | **3.19×10^−83^** |
|  |  | 1 | 0.101 | 0.0052 | 0.0101 | **2.56×10^−82^** |
|  | Years of Education | 0.01 | 0.077 | 0.0052 | 0.0058 | **1.88×10^−48^** |
|  |  | 0.05 | 0.090 | 0.0052 | 0.0080 | **2.26×10^−65^** |
|  |  | 0.1 | 0.096 | 0.0052 | 0.0091 | **7.36×10^−75^** |
|  |  | 0.5 | 0.105 | 0.0053 | 0.0109 | **6.20×10^−89^** |
|  |  | 1 | 0.106 | 0.0053 | 0.0109 | **2.96×10^−89^** |

**Supplementary Table 4b**

Associations between polygenic profiles of health related traits and Reaction Time (log transformed) controlling for age, sex, assessment centre, genotyping batch and array, and ten principal components for population structure. Statistically significant values (P<0.0188) are shown in bold. Cognitive/education phenotypes are scored such that higher scores indicate better performance.

| **Trait category** | **Trait** | **Reaction Time** | | | | |
| --- | --- | --- | --- | --- | --- | --- |
|  |  | Threshold | β | SE | R2 | p |
| **Vascular-Metabolic Diseases** | Coronary Artery Disease | 0.01 | 3.24×10^−5^ | 0.0005 | 3.03×10^−8^ | 0.9508 |
|  |  | 0.05 | 2.80×10^−4^ | 0.0005 | 2.27×10^−6^ | 0.5939 |
|  |  | 0.1 | 0.001 | 0.0005 | 2.33×10^−5^ | 0.0875 |
|  |  | 0.5 | 0.001 | 0.0005 | 1.37×10^−5^ | 0.1894 |
|  |  | 1 | 0.001 | 0.0005 | 1.83×10^−5^ | 0.1301 |
|  | Stroke: Ischaemic | 0.01 | −0.001 | 0.0005 | 2.46×10^−5^ | 0.0791 |
|  |  | 0.05 | −3.00×10^−4^ | 0.0005 | 2.53×10^−6^ | 0.5735 |
|  |  | 0.1 | 2.52×10^−4^ | 0.0005 | 1.76×10^−6^ | 0.6385 |
|  |  | 0.5 | −3.68×10^−5^ | 0.0005 | 3.73×10^−8^ | 0.9455 |
|  |  | 1 | −1.53×10^−4^ | 0.0005 | 6.58×10^−7^ | 0.7740 |
|  | Stroke: Cardioembolic | 0.01 | 0.001 | 0.0005 | 2.42×10^−5^ | 0.0817 |
|  |  | 0.05 | 0.001 | 0.0005 | 1.47×10^−5^ | 0.1746 |
|  |  | 0.1 | 0.001 | 0.0005 | 7.98×10^−6^ | 0.3175 |
|  |  | 0.5 | 1.74×10^−4^ | 0.0005 | 8.71×10^−7^ | 0.7412 |
|  |  | 1 | −2.32×10^−5^ | 0.0005 | 1.55×10^−8^ | 0.9648 |
|  | Stroke: Large Vessel Disease | 0.01 | 5.86×10^−5^ | 0.0005 | 9.86×10^−8^ | 0.9115 |
|  |  | 0.05 | 2.26×10^−4^ | 0.0005 | 1.44×10^−6^ | 0.6714 |
|  |  | 0.1 | −1.54×10^−4^ | 0.0005 | 6.66×10^−7^ | 0.7726 |
|  |  | 0.5 | 4.45×10^−6^ | 0.0005 | 5.72×10^−10^ | 0.9932 |
|  |  | 1 | −2.03×10^−4^ | 0.0005 | 1.19×10^−6^ | 0.6991 |
|  | Stroke: Small Vessel Disease | 0.01 | −3.12×10^−4^ | 0.0005 | 2.82×10^−6^ | 0.5525 |
|  |  | 0.05 | 1.05×10^−4^ | 0.0005 | 3.16×10^−7^ | 0.8424 |
|  |  | 0.1 | −1.45×10^−4^ | 0.0005 | 6.04×10^−7^ | 0.7832 |
|  |  | 0.5 | −4.53×10^−4^ | 0.0005 | 5.94×10^−6^ | 0.3882 |
|  |  | 1 | −0.001 | 0.0005 | 7.93×10^−6^ | 0.3188 |
|  | Type 2 Diabetes | 0.01 | −4.33×10^−4^ | 0.0005 | 5.36×10^−6^ | 0.4124 |
|  |  | 0.05 | −3.10×10^−4^ | 0.0005 | 2.71×10^−6^ | 0.5602 |
|  |  | 0.1 | −4.50×10^−4^ | 0.0005 | 5.70×10^−6^ | 0.3982 |
|  |  | 0.5 | −3.65×10^−5^ | 0.0005 | 3.67×10^−8^ | 0.9459 |
|  |  | 1 | −8.44×10^−5^ | 0.0005 | 1.96×10^−7^ | 0.8755 |
| **Neuro-Psychiatric Disorders** | ADHD | 0.01 | −2.71×10^−4^ | 0.0005 | 2.13×10^−6^ | 0.6053 |
|  |  | 0.05 | 6.76×10^−5^ | 0.0005 | 1.32×10^−7^ | 0.8976 |
|  |  | 0.1 | 4.14×10^−5^ | 0.0005 | 4.97×10^−8^ | 0.9371 |
|  |  | 0.5 | −0.001 | 0.0005 | 8.45×10^−6^ | 0.3036 |
|  |  | 1 | −0.001 | 0.0005 | 1.06×10^−5^ | 0.2485 |
|  | Alzheimer's Disease | 0.01 | −4.31×10^−4^ | 0.0005 | 5.38×10^−6^ | 0.4116 |
|  |  | 0.05 | −0.001 | 0.0005 | 2.04×10^−5^ | 0.1096 |
|  |  | 0.1 | −2.30×10^−4^ | 0.0005 | 1.53×10^−6^ | 0.6619 |
|  |  | 0.5 | −0.001 | 0.0005 | 3.02×10^−5^ | 0.0516 |
|  |  | 1 | −0.001 | 0.0005 | 2.64×10^−5^ | 0.0692 |
|  | Autism | 0.01 | 0.001 | 0.0005 | 1.78×10^−5^ | 0.1348 |
|  |  | 0.05 | 0.001 | 0.0005 | 1.24×10^−5^ | 0.2124 |
|  |  | 0.1 | 0.001 | 0.0005 | 1.95×10^−5^ | 0.1176 |
|  |  | 0.5 | 0.001 | 0.0005 | 1.01×10^−5^ | 0.2612 |
|  |  | 1 | 0.001 | 0.0005 | 1.01×10^−5^ | 0.2605 |
|  | Bipolar Disorder | 0.01 | −0.002 | 0.0005 | 9.08×10^−5^ | **0.0007** |
|  |  | 0.05 | −0.002 | 0.0005 | 7.79×10^−5^ | **0.0018** |
|  |  | 0.1 | −0.002 | 0.0005 | 8.68×10^−5^ | **0.0010** |
|  |  | 0.5 | −0.002 | 0.0005 | 8.54×10^−5^ | **0.0011** |
|  |  | 1 | −0.002 | 0.0005 | 8.98×10^−5^ | **0.0008** |
|  | Major Depressive Disorder | 0.01 | −0.001 | 0.0005 | 2.95×10^−5^ | 0.0544 |
|  |  | 0.05 | −0.002 | 0.0005 | 7.22×10^−5^ | **0.0026** |
|  |  | 0.1 | −0.002 | 0.0005 | 9.61×10^−5^ | **0.0005** |
|  |  | 0.5 | −0.002 | 0.0005 | 8.30×10^−5^ | **0.0013** |
|  |  | 1 | −0.002 | 0.0005 | 6.50×10^−5^ | **0.0043** |
|  | Schizophrenia | 0.01 | −0.005 | 0.0005 | 0.0007 | **4.24×10^−22^** |
|  |  | 0.05 | −0.005 | 0.0005 | 0.0008 | **1.03×10^−23^** |
|  |  | 0.1 | −0.006 | 0.0005 | 0.0009 | **1.88×10^−25^** |
|  |  | 0.5 | −0.005 | 0.0005 | 0.0008 | **7.56×10^−24^** |
|  |  | 1 | −0.005 | 0.0005 | 0.0008 | **2.38×10^−23^** |
| **Brain Measures** | Hippocampal Volume | 0.01 | 0.001 | 0.0005 | 8.02×10^−6^ | 0.3163 |
|  |  | 0.05 | 0.001 | 0.0005 | 1.47×10^−5^ | 0.1744 |
|  |  | 0.1 | 0.001 | 0.0005 | 1.68×10^−5^ | 0.1465 |
|  |  | 0.5 | 0.001 | 0.0005 | 1.99×10^−5^ | 0.1144 |
|  |  | 1 | 0.001 | 0.0005 | 1.98×10^−5^ | 0.1156 |
|  | Intracranial Volume | 0.01 | 0.001 | 0.0005 | 7.56×10^−6^ | 0.3306 |
|  |  | 0.05 | 3.57×10^−4^ | 0.0005 | 3.60×10^−6^ | 0.5020 |
|  |  | 0.1 | 3.32×10^−4^ | 0.0005 | 3.08×10^−6^ | 0.5346 |
|  |  | 0.5 | 3.29×10^−4^ | 0.0005 | 2.97×10^−6^ | 0.5418 |
|  |  | 1 | 3.21×10^−4^ | 0.0005 | 2.83×10^−6^ | 0.5514 |
|  | Infant Head Circumference | 0.01 | −0.001 | 0.0005 | 3.16×10^−5^ | 0.0468 |
|  |  | 0.05 | −3.01×10^−4^ | 0.0005 | 2.60×10^−6^ | 0.5683 |
|  |  | 0.1 | −1.06×10^−4^ | 0.0005 | 3.20×10^−7^ | 0.8413 |
|  |  | 0.5 | −1.92×10^−4^ | 0.0005 | 1.05×10^−6^ | 0.7174 |
|  |  | 1 | −2.02×10^−4^ | 0.0005 | 1.15×10^−6^ | 0.7037 |
| **Physical and physiological measures** | Blood Pressure: Diastolic | 0.01 | 0.001 | 0.0005 | 1.14×10^−5^ | 0.2325 |
|  |  | 0.05 | 1.59×10^−4^ | 0.0005 | 7.28×10^−7^ | 0.7627 |
|  |  | 0.1 | 3.45×10^−4^ | 0.0005 | 3.43×10^−6^ | 0.5120 |
|  |  | 0.5 | 4.50×10^−4^ | 0.0005 | 5.82×10^−6^ | 0.3931 |
|  |  | 1 | 0.001 | 0.0005 | 7.61×10^−6^ | 0.3289 |
|  | Blood Pressure: Systolic | 0.01 | −1.73×10^−4^ | 0.0005 | 8.68×10^−7^ | 0.7416 |
|  |  | 0.05 | −2.58×10^−4^ | 0.0005 | 1.92×10^−6^ | 0.6241 |
|  |  | 0.1 | −4.81×10^−4^ | 0.0005 | 6.66×10^−6^ | 0.3609 |
|  |  | 0.5 | −4.97×10^−4^ | 0.0005 | 7.09×10^−6^ | 0.3460 |
|  |  | 1 | −3.88×10^−4^ | 0.0005 | 4.32×10^−6^ | 0.4619 |
|  | BMI | 0.01 | 3.96×10^−4^ | 0.0005 | 4.54×10^−6^ | 0.4507 |
|  |  | 0.05 | 1.99×10^−5^ | 0.0005 | 1.15×10^−8^ | 0.9697 |
|  |  | 0.1 | −4.02×10^−5^ | 0.0005 | 4.67×10^−8^ | 0.9390 |
|  |  | 0.5 | −2.47×10^−5^ | 0.0005 | 1.76×10^−8^ | 0.9626 |
|  |  | 1 | 1.79×10^−5^ | 0.0005 | 9.25×10^−9^ | 0.9728 |
|  | Height | 0.01 | 3.64×10^−4^ | 0.0005 | 3.75×10^−6^ | 0.4933 |
|  |  | 0.05 | 2.82×10^−4^ | 0.0005 | 2.21×10^−6^ | 0.5985 |
|  |  | 0.1 | 4.06×10^−4^ | 0.0005 | 4.54×10^−6^ | 0.4508 |
|  |  | 0.5 | 4.46×10^−4^ | 0.0005 | 5.34×10^−6^ | 0.4135 |
|  |  | 1 | 4.07×10^−4^ | 0.0005 | 4.43×10^−6^ | 0.4562 |
|  | Longevity | 0.01 | 2.47×10^−5^ | 0.0005 | 1.75×10^−8^ | 0.9627 |
|  |  | 0.05 | 3.23×10^−4^ | 0.0005 | 2.96×10^−6^ | 0.5426 |
|  |  | 0.1 | 1.21×10^−4^ | 0.0005 | 4.25×10^−7^ | 0.8176 |
|  |  | 0.5 | 2.30×10^−4^ | 0.0005 | 1.52×10^−6^ | 0.6623 |
|  |  | 1 | 1.84×10^−4^ | 0.0005 | 9.73×10^−7^ | 0.7270 |
|  | Forced Expiratory Volume in 1s (FEV_1_) | 0.01 | −4.27×10^−4^ | 0.0005 | 5.28×10^−6^ | 0.4162 |
|  |  | 0.05 | −0.001 | 0.0005 | 1.83×10^−5^ | 0.1302 |
|  |  | 0.1 | −0.001 | 0.0005 | 1.05×10^−5^ | 0.2505 |
|  |  | 0.5 | −2.14×10^−4^ | 0.0005 | 1.33×10^−6^ | 0.6836 |
|  |  | 1 | −2.52×10^−4^ | 0.0005 | 1.84×10^−6^ | 0.6310 |
| **Life-course Cognitive Traits and Proxies** | Childhood Cognitive Ability | 0.01 | 0.001 | 0.0005 | 1.57×10^−5^ | 0.1613 |
|  |  | 0.05 | 0.002 | 0.0005 | 8.41×10^−5^ | **0.0012** |
|  |  | 0.1 | 0.002 | 0.0005 | 9.30×10^−5^ | **0.0006** |
|  |  | 0.5 | 0.002 | 0.0005 | 0.0001 | **0.0002** |
|  |  | 1 | 0.002 | 0.0005 | 8.94×10^−5^ | **0.0008** |
|  | College Degree | 0.01 | 0.001 | 0.0005 | 1.60×10^−5^ | 0.1573 |
|  |  | 0.05 | 0.001 | 0.0005 | 4.61×10^−5^ | **0.0163** |
|  |  | 0.1 | 0.001 | 0.0005 | 3.97×10^−5^ | 0.0257 |
|  |  | 0.5 | 0.001 | 0.0005 | 4.90×10^−5^ | **0.0133** |
|  |  | 1 | 0.001 | 0.0005 | 4.42×10^−5^ | **0.0186** |
|  | Years of Education | 0.01 | 0.001 | 0.0005 | 2.26×10^−5^ | 0.0927 |
|  |  | 0.05 | 0.001 | 0.0005 | 2.15×10^−5^ | 0.1008 |
|  |  | 0.1 | 0.001 | 0.0005 | 4.67×10^−5^ | **0.0156** |
|  |  | 0.5 | 0.001 | 0.0005 | 2.71×10^−5^ | 0.0654 |
|  |  | 1 | 0.001 | 0.0005 | 2.92×10^−5^ | 0.0558 |

**Supplementary Table 4c**

Associations between polygenic profiles of health related traits, and memory controlling for age, sex, assessment centre, genotyping batch and array, and ten principal components for population structure. Statistically significant values (P<0**.**0188) are shown in bold. Cognitive/education phenotypes are scored such that higher scores indicate better performance.

| **Trait category** | **Trait** | **Memory** | | | | |
| --- | --- | --- | --- | --- | --- | --- |
|  |  | Threshold | β | SE | R2 | p |
| **Vascular-Metabolic Diseases** | Coronary Artery Disease | 0.01 | −0.003 | 0.0030 | 1.20×10^−5^ | 0.2399 |
|  |  | 0.05 | −0.002 | 0.0030 | 5.65×10^−6^ | 0.4207 |
|  |  | 0.1 | −0.0003 | 0.0030 | 8.02×10^−8^ | 0.9236 |
|  |  | 0.5 | 0.0003 | 0.0030 | 1.17×10^−7^ | 0.9079 |
|  |  | 1 | 0.001 | 0.0030 | 3.17×10^−7^ | 0.8487 |
|  | Stroke: Ischaemic | 0.01 | −0.002 | 0.0030 | 3.97×10^−6^ | 0.4995 |
|  |  | 0.05 | −0.001 | 0.0030 | 4.35×10^−7^ | 0.8231 |
|  |  | 0.1 | −0.0005 | 0.0030 | 2.23×10^−7^ | 0.8728 |
|  |  | 0.5 | −0.004 | 0.0030 | 1.58×10^−5^ | 0.1782 |
|  |  | 1 | −0.003 | 0.0030 | 1.12×10^−5^ | 0.2562 |
|  | Stroke: Cardioembolic | 0.01 | 0.004 | 0.0030 | 1.43×10^−5^ | 0.2002 |
|  |  | 0.05 | −0.001 | 0.0030 | 9.14×10^−7^ | 0.7460 |
|  |  | 0.1 | −0.004 | 0.0030 | 1.39×10^−5^ | 0.2071 |
|  |  | 0.5 | 0.004 | 0.0030 | 1.52×10^−5^ | 0.1870 |
|  |  | 1 | 0.005 | 0.0030 | 2.85×10^−5^ | 0.0707 |
|  | Stroke: Large Vessel Disease | 0.01 | −0.005 | 0.0030 | 2.91×10^−5^ | 0.0678 |
|  |  | 0.05 | −0.006 | 0.0030 | 3.15×10^−5^ | 0.0573 |
|  |  | 0.1 | −0.008 | 0.0030 | 6.89×10^−5^ | **0.0049** |
|  |  | 0.5 | 0.0001 | 0.0030 | 4.07×10^−9^ | 0.9828 |
|  |  | 1 | −0.001 | 0.0030 | 1.55×10^−6^ | 0.6727 |
|  | Stroke: Small Vessel Disease | 0.01 | −0.005 | 0.0030 | 2.12×10^−5^ | 0.1191 |
|  |  | 0.05 | −0.004 | 0.0030 | 1.54×10^−5^ | 0.1834 |
|  |  | 0.1 | −0.003 | 0.0030 | 7.26×10^−6^ | 0.3612 |
|  |  | 0.5 | −0.004 | 0.0030 | 1.59×10^−5^ | 0.1765 |
|  |  | 1 | −0.003 | 0.0030 | 1.19×10^−5^ | 0.2431 |
|  | Type 2 Diabetes | 0.01 | 0.0002 | 0.0030 | 2.52×10^−8^ | 0.9572 |
|  |  | 0.05 | 0.002 | 0.0030 | 4.55×10^−6^ | 0.4697 |
|  |  | 0.1 | 0.001 | 0.0030 | 2.81×10^−7^ | 0.8575 |
|  |  | 0.5 | 0.003 | 0.0030 | 8.32×10^−6^ | 0.3285 |
|  |  | 1 | 0.003 | 0.0030 | 1.12×10^−5^ | 0.2562 |
| **Neuro-Psychiatric Disorders** | ADHD | 0.01 | 0.0001 | 0.0030 | 1.01×10^−8^ | 0.9728 |
|  |  | 0.05 | −0.001 | 0.0030 | 4.60×10^−7^ | 0.8184 |
|  |  | 0.1 | 0.001 | 0.0030 | 7.67×10^−7^ | 0.7667 |
|  |  | 0.5 | 0.001 | 0.0030 | 1.78×10^−6^ | 0.6511 |
|  |  | 1 | 0.001 | 0.0030 | 2.61×10^−7^ | 0.8627 |
|  | Alzheimer's Disease | 0.01 | −0.009 | 0.0030 | 7.37×10^−5^ | **0.0036** |
|  |  | 0.05 | −0.009 | 0.0030 | 7.37×10^−5^ | **0.0036** |
|  |  | 0.1 | −0.011 | 0.0030 | 1.26×10^−4^ | **0.0001** |
|  |  | 0.5 | −0.010 | 0.0030 | 9.05×10^−5^ | **0.0013** |
|  |  | 1 | −0.009 | 0.0030 | 8.66×10^−5^ | **0.0016** |
|  | Autism | 0.01 | 0.002 | 0.0030 | 2.30×10^−6^ | 0.6072 |
|  |  | 0.05 | 0.001 | 0.0030 | 1.31×10^−6^ | 0.6983 |
|  |  | 0.1 | 0.003 | 0.0030 | 7.77×10^−6^ | 0.3450 |
|  |  | 0.5 | −0.001 | 0.0030 | 1.00×10^−6^ | 0.7345 |
|  |  | 1 | −0.001 | 0.0030 | 1.43×10^−6^ | 0.6856 |
|  | Bipolar Disorder | 0.01 | −0.013 | 0.0030 | 1.56×10^−4^ | **2.27×10^−5^** |
|  |  | 0.05 | −0.015 | 0.0030 | 2.12×10^−4^ | **8.12×10^−7^** |
|  |  | 0.1 | −0.015 | 0.0030 | 2.30×10^−4^ | **2.70×10^−7^** |
|  |  | 0.5 | −0.017 | 0.0030 | 2.74×10^−4^ | **2.02×10^−8^** |
|  |  | 1 | −0.016 | 0.0030 | 2.39×10^−4^ | **1.63×10^−7^** |
|  | Major Depressive Disorder | 0.01 | −0.007 | 0.0030 | 4.88×10^−5^ | **0.0179** |
|  |  | 0.05 | −0.006 | 0.0030 | 3.68×10^−5^ | 0.0398 |
|  |  | 0.1 | −0.010 | 0.0030 | 9.61×10^−5^ | **0.0009** |
|  |  | 0.5 | −0.013 | 0.0030 | 1.63×10^−4^ | **1.49×10^−5^** |
|  |  | 1 | −0.014 | 0.0030 | 1.87×10^−4^ | **3.67×10^−6^** |
|  | Schizophrenia | 0.01 | −0.035 | 0.0030 | 1.20×10^−3^ | **8.91×10^−32^** |
|  |  | 0.05 | −0.039 | 0.0030 | 1.50×10^−3^ | **1.98×10^−3^9** |
|  |  | 0.1 | −0.040 | 0.0030 | 1.52×10^−3^ | **9.27×10^−40^** |
|  |  | 0.5 | −0.038 | 0.0030 | 1.37×10^−3^ | **3.38×10^−36^** |
|  |  | 1 | −0.038 | 0.0030 | 1.32×10^−3^ | **6.33×10^−35^** |
| **Brain Measures** | Hippocampal Volume | 0.01 | −0.003 | 0.0030 | 7.55×10^−6^ | 0.3520 |
|  |  | 0.05 | −0.003 | 0.0030 | 1.15×10^−5^ | 0.2504 |
|  |  | 0.1 | −0.005 | 0.0030 | 2.65×10^−5^ | 0.0812 |
|  |  | 0.5 | −0.003 | 0.0030 | 7.42×10^−6^ | 0.3561 |
|  |  | 1 | −0.003 | 0.0030 | 1.04×10^−5^ | 0.2756 |
|  | Intracranial Volume | 0.01 | 0.0003 | 0.0030 | 1.01×10^−7^ | 0.9141 |
|  |  | 0.05 | 0.001 | 0.0030 | 3.92×10^−7^ | 0.8319 |
|  |  | 0.1 | 0.004 | 0.0030 | 1.67×10^−5^ | 0.1660 |
|  |  | 0.5 | 0.005 | 0.0030 | 2.63×10^−5^ | 0.0821 |
|  |  | 1 | 0.005 | 0.0030 | 2.77×10^−5^ | 0.0745 |
|  | Infant Head Circumference | 0.01 | 0.002 | 0.0030 | 4.06×10^−6^ | 0.4947 |
|  |  | 0.05 | 0.001 | 0.0030 | 1.45×10^−6^ | 0.6830 |
|  |  | 0.1 | 0.005 | 0.0030 | 2.78×10^−5^ | 0.0742 |
|  |  | 0.5 | 0.003 | 0.0030 | 8.28×10^−6^ | 0.3297 |
|  |  | 1 | 0.002 | 0.0030 | 4.78×10^−6^ | 0.4588 |
| **Physical and physiological measures** | Blood Pressure: Diastolic | 0.01 | 0.002 | 0.0030 | 4.34×10^−6^ | 0.4803 |
|  |  | 0.05 | −0.0004 | 0.0030 | 1.41×10^−7^ | 0.8986 |
|  |  | 0.1 | 0.001 | 0.0030 | 1.32×10^−6^ | 0.6968 |
|  |  | 0.5 | −0.001 | 0.0030 | 1.68×10^−6^ | 0.6607 |
|  |  | 1 | −0.002 | 0.0030 | 2.90×10^−6^ | 0.5638 |
|  | Blood Pressure: Systolic | 0.01 | −0.002 | 0.0030 | 4.60×10^−6^ | 0.4674 |
|  |  | 0.05 | −0.003 | 0.0030 | 7.37×10^−6^ | 0.3578 |
|  |  | 0.1 | −0.002 | 0.0030 | 2.34×10^−6^ | 0.6039 |
|  |  | 0.5 | −0.003 | 0.0030 | 7.00×10^−6^ | 0.3699 |
|  |  | 1 | −0.002 | 0.0030 | 3.99×10^−6^ | 0.4985 |
|  | BMI | 0.01 | 0.009 | 0.0030 | 7.54×10^−5^ | **0.0033** |
|  |  | 0.05 | 0.013 | 0.0030 | 1.68×10^−4^ | **1.16×10^−5^** |
|  |  | 0.1 | 0.016 | 0.0030 | 2.60×10^−4^ | **4.76×10^−8^** |
|  |  | 0.5 | 0.014 | 0.0030 | 1.97×10^−4^ | **1.96×10^−6^** |
|  |  | 1 | 0.014 | 0.0030 | 1.97×10^−4^ | **1.99×10^−6^** |
|  | Height | 0.01 | 0.003 | 0.0030 | 7.70×10^−6^ | 0.3472 |
|  |  | 0.05 | 0.001 | 0.0030 | 1.76×10^−6^ | 0.6534 |
|  |  | 0.1 | 0.002 | 0.0030 | 2.41×10^−6^ | 0.5991 |
|  |  | 0.5 | 0.001 | 0.0031 | 1.90×10^−6^ | 0.6407 |
|  |  | 1 | 0.001 | 0.0031 | 9.21×10^−7^ | 0.7451 |
|  | Longevity | 0.01 | 0.004 | 0.0030 | 1.66×10^−5^ | 0.1680 |
|  |  | 0.05 | 0.003 | 0.0030 | 1.13×10^−5^ | 0.2539 |
|  |  | 0.1 | 0.004 | 0.0030 | 1.73×10^−5^ | 0.1583 |
|  |  | 0.5 | 0.005 | 0.0030 | 2.24×10^−5^ | 0.1087 |
|  |  | 1 | 0.005 | 0.0030 | 2.88×10^−5^ | 0.0689 |
|  | Forced Expiratory Volume in 1s (FEV_1_) | 0.01 | −0.005 | 0.0030 | 2.40×10^−5^ | 0.0968 |
|  |  | 0.05 | −0.005 | 0.0030 | 2.60×10^−5^ | 0.0840 |
|  |  | 0.1 | −0.005 | 0.0030 | 2.04×10^−5^ | 0.1256 |
|  |  | 0.5 | −0.005 | 0.0030 | 2.48×10^−5^ | 0.0917 |
|  |  | 1 | −0.005 | 0.0030 | 2.66×10^−5^ | 0.0806 |
| **Life-course Cognitive Traits and Proxies** | Childhood Cognitive Ability | 0.01 | 0.003 | 0.0030 | 1.21×10^−5^ | 0.2395 |
|  |  | 0.05 | 0.006 | 0.0030 | 3.23×10^−5^ | 0.0543 |
|  |  | 0.1 | 0.007 | 0.0030 | 5.49×10^−5^ | **0.0121** |
|  |  | 0.5 | 0.014 | 0.0030 | 1.87×10^−4^ | **3.54×10^−6^** |
|  |  | 1 | 0.014 | 0.0030 | 2.03×10^−4^ | **1.38×10^−6^** |
|  | College degree | 0.01 | 0.0002 | 0.0030 | 2.98×10^−8^ | 0.9534 |
|  |  | 0.05 | −0.002 | 0.0030 | 5.65×10^−6^ | 0.4207 |
|  |  | 0.1 | −0.002 | 0.0030 | 5.32×10^−6^ | 0.4344 |
|  |  | 0.5 | 0.001 | 0.0030 | 1.32×10^−6^ | 0.6970 |
|  |  | 1 | 0.002 | 0.0030 | 2.26×10^−6^ | 0.6106 |
|  | Years of Education | 0.01 | −0.0005 | 0.0030 | 2.11×10^−7^ | 0.8765 |
|  |  | 0.05 | −0.004 | 0.0030 | 1.81×10^−5^ | 0.1496 |
|  |  | 0.1 | −0.004 | 0.0030 | 1.22×10^−5^ | 0.2358 |
|  |  | 0.5 | −0.002 | 0.0030 | 6.11×10^−6^ | 0.4022 |
|  |  | 1 | −0.002 | 0.0030 | 2.69×10^−6^ | 0.5787 |

**Supplementary Table 4d**

Associations between polygenic profiles of health related traits, and college controlling for age, sex, assessment centre, genotyping batch and array, and ten principal components for population structure. Statistically significant values (P<0**.**0188) are shown in bold. Cognitive/education phenotypes are scored such that higher scores indicate better performance.

| **Trait category** | **Trait** | **Educational Attainment** | | | | |
| --- | --- | --- | --- | --- | --- | --- |
|  |  | Threshold | β | SE | R2 | p |
| **Vascular-Metabolic Diseases** | Coronary Artery Disease | 0.01 | −0.020 | 0.0066 | 0.0001 | **0.0020** |
|  |  | 0.05 | −0.026 | 0.0066 | 0.0002 | **0.0001** |
|  |  | 0.1 | −0.033 | 0.0066 | 0.0003 | **8.09×10^−7^** |
|  |  | 0.5 | −0.047 | 0.0066 | 0.0006 | **8.32×10^−13^** |
|  |  | 1 | −0.047 | 0.0066 | 0.0006 | **7.92×10^−13^** |
|  | Stroke: Ischaemic | 0.01 | −0.013 | 0.0066 | 4.64×10^−5^ | 0.0539 |
|  |  | 0.05 | −0.020 | 0.0067 | 0.0001 | **0.0032** |
|  |  | 0.1 | −0.016 | 0.0067 | 0.0001 | **0.0171** |
|  |  | 0.5 | −0.020 | 0.0068 | 0.0001 | **0.0026** |
|  |  | 1 | −0.013 | 0.0068 | 4.67×10^−5^ | 0.0534 |
|  | Stroke: Cardioembolic | 0.01 | −0.0001 | 0.0066 | 6.41×10^−9^ | 0.9819 |
|  |  | 0.05 | −0.001 | 0.0066 | 1.43×10^−7^ | 0.9148 |
|  |  | 0.1 | 0.001 | 0.0066 | 2.76×10^−7^ | 0.8817 |
|  |  | 0.5 | −0.003 | 0.0066 | 1.94×10^−6^ | 0.6931 |
|  |  | 1 | 0.000 | 0.0066 | 4.94×10^−1^2 | 0.9995 |
|  | Stroke: Large Vessel Disease | 0.01 | −0.023 | 0.0066 | 0.0002 | **0.0005** |
|  |  | 0.05 | −0.025 | 0.0067 | 0.0002 | **0.0001** |
|  |  | 0.1 | −0.031 | 0.0067 | 0.0003 | **5.31×10^−6^** |
|  |  | 0.5 | 0.008 | 0.0066 | 1.70×10^−5^ | 0.2438 |
|  |  | 1 | 0.001 | 0.0066 | 5.27×10^−7^ | 0.8372 |
|  | Stroke: Small Vessel Disease | 0.01 | −0.024 | 0.0066 | 0.0002 | **0.0003** |
|  |  | 0.05 | −0.030 | 0.0066 | 0.0003 | **5.89×10^−6^** |
|  |  | 0.1 | −0.034 | 0.0066 | 0.0003 | **2.96×10^−7^** |
|  |  | 0.5 | 0.000 | 0.0066 | 3.41×10^−8^ | 0.9583 |
|  |  | 1 | 0.002 | 0.0066 | 1.15×10^−6^ | 0.7615 |
|  | Type 2 Diabetes | 0.01 | −0.023 | 0.0066 | 0.0001 | **0.0006** |
|  |  | 0.05 | −0.021 | 0.0067 | 0.0001 | **0.0020** |
|  |  | 0.1 | −0.025 | 0.0067 | 0.0002 | **0.0002** |
|  |  | 0.5 | −0.025 | 0.0067 | 0.0002 | **0.0002** |
|  |  | 1 | −0.024 | 0.0068 | 0.0002 | **0.0003** |
| **Neuro-Psychiatric Disorders** | ADHD | 0.01 | −0.010 | 0.0066 | 2.73×10^−5^ | 0.1391 |
|  |  | 0.05 | −0.016 | 0.0066 | 0.0001 | **0.0138** |
|  |  | 0.1 | −0.022 | 0.0066 | 0.0001 | **0.0007** |
|  |  | 0.5 | −0.025 | 0.0066 | 0.0002 | **0.0001** |
|  |  | 1 | −0.027 | 0.0066 | 0.0002 | **4.68×10^−5^** |
|  | Alzheimer's Disease | 0.01 | −0.013 | 0.0066 | 4.51×10^−5^ | 0.0572 |
|  |  | 0.05 | −0.034 | 0.0066 | 0.0003 | **2.53×10^−7^** |
|  |  | 0.1 | −0.038 | 0.0066 | 0.0004 | **7.00×10^−9^** |
|  |  | 0.5 | −0.046 | 0.0066 | 0.0006 | **2.52×10^−12^** |
|  |  | 1 | −0.046 | 0.0066 | 0.0006 | **2.33×10^−12^** |
|  | Autism | 0.01 | 0.046 | 0.0066 | 0.0006 | **4.09×10^−12^** |
|  |  | 0.05 | 0.061 | 0.0066 | 0.0011 | **1.68×10^−20^** |
|  |  | 0.1 | 0.060 | 0.0066 | 0.0010 | **1.42×10^−19^** |
|  |  | 0.5 | 0.068 | 0.0066 | 0.0013 | **5.00×10^−25^** |
|  |  | 1 | 0.067 | 0.0066 | 0.0013 | **2.88×10^−24^** |
|  | Bipolar Disorder | 0.01 | 0.032 | 0.0066 | 0.0003 | **9.40×10^−7^** |
|  |  | 0.05 | 0.046 | 0.0066 | 0.0006 | **3.80×10^−12^** |
|  |  | 0.1 | 0.050 | 0.0066 | 0.0007 | **3.41×10^−14^** |
|  |  | 0.5 | 0.057 | 0.0067 | 0.0009 | **1.35×10^−17^** |
|  |  | 1 | 0.055 | 0.0067 | 0.0008 | **2.60×10^−16^** |
|  | Major Depressive Disorder | 0.01 | 0.004 | 0.0066 | 4.03×10^−6^ | 0.5700 |
|  |  | 0.05 | 0.003 | 0.0066 | 2.61×10^−6^ | 0.6475 |
|  |  | 0.1 | 0.003 | 0.0067 | 2.22×10^−6^ | 0.6731 |
|  |  | 0.5 | −0.006 | 0.0067 | 1.14×10^−5^ | 0.3400 |
|  |  | 1 | −0.009 | 0.0067 | 2.14×10^−5^ | 0.1905 |
|  | Schizophrenia | 0.01 | 0.022 | 0.0066 | 0.0001 | **0.0008** |
|  |  | 0.05 | 0.025 | 0.0067 | 0.0002 | **0.0002** |
|  |  | 0.1 | 0.024 | 0.0067 | 0.0002 | **0.0004** |
|  |  | 0.5 | 0.022 | 0.0068 | 0.0001 | **0.0012** |
|  |  | 1 | 0.021 | 0.0068 | 0.0001 | **0.0017** |
| **Brain Measures** | Hippocampal Volume | 0.01 | −0.007 | 0.0066 | 1.27×10^−5^ | 0.3132 |
|  |  | 0.05 | −0.001 | 0.0066 | 6.01×10^−7^ | 0.8262 |
|  |  | 0.1 | 0.005 | 0.0066 | 6.29×10^−6^ | 0.4778 |
|  |  | 0.5 | 0.007 | 0.0066 | 1.40×10^−5^ | 0.2887 |
|  |  | 1 | 0.005 | 0.0066 | 8.49×10^−6^ | 0.4095 |
|  | Intracranial Volume | 0.01 | 0.031 | 0.0066 | 2.81×10^−4^ | **2.08×10^−6^** |
|  |  | 0.05 | 0.034 | 0.0067 | 0.0003 | **4.30×10^−7^** |
|  |  | 0.1 | 0.039 | 0.0067 | 0.0004 | **7.92×10^−9^** |
|  |  | 0.5 | 0.041 | 0.0068 | 0.0005 | **8.86×10^−10^** |
|  |  | 1 | 0.043 | 0.0068 | 0.0005 | **2.91×10^−10^** |
|  | Infant Head Circumference | 0.01 | 0.035 | 0.0066 | 0.0003 | **1.64×10^−7^** |
|  |  | 0.05 | 0.038 | 0.0066 | 0.0004 | **6.43×10^−9^** |
|  |  | 0.1 | 0.050 | 0.0066 | 0.0007 | **4.01×10^−14^** |
|  |  | 0.5 | 0.049 | 0.0067 | 0.0007 | **1.37×10^−13^** |
|  |  | 1 | 0.049 | 0.0067 | 0.0007 | **2.32×10^−13^** |
| **Physical and physiological measures** | Blood Pressure: Diastolic | 0.01 | 0.004 | 0.0066 | 5.75×10^−6^ | 0.4971 |
|  |  | 0.05 | 0.020 | 0.0066 | 0.0001 | **0.0022** |
|  |  | 0.1 | 0.022 | 0.0066 | 0.0001 | **0.0011** |
|  |  | 0.5 | 0.020 | 0.0066 | 0.0001 | **0.0028** |
|  |  | 1 | 0.018 | 0.0066 | 0.0001 | **0.0053** |
|  | Blood Pressure: Systolic | 0.01 | 0.017 | 0.0066 | 0.0001 | **0.0095** |
|  |  | 0.05 | 0.032 | 0.0066 | 0.0003 | **8.25×10^−7^** |
|  |  | 0.1 | 0.035 | 0.0066 | 0.0004 | **7.61×10^−8^** |
|  |  | 0.5 | 0.027 | 0.0066 | 0.0002 | **4.66×10^−5^** |
|  |  | 1 | 0.027 | 0.0066 | 0.0002 | **4.35×10^−5^** |
|  | BMI | 0.01 | −0.065 | 0.0066 | 0.0012 | **3.15×10^−23^** |
|  |  | 0.05 | −0.074 | 0.0066 | 0.0016 | **2.80×10^−29^** |
|  |  | 0.1 | −0.081 | 0.0066 | 0.0019 | **7.97×10^−35^** |
|  |  | 0.5 | −0.093 | 0.0066 | 0.0025 | **6.40×10^−45^** |
|  |  | 1 | −0.093 | 0.0066 | 0.0025 | **1.07×10^−44^** |
|  | Height | 0.01 | 0.054 | 0.0067 | 0.0008 | **8.35×10^−16^** |
|  |  | 0.05 | 0.062 | 0.0067 | 0.0011 | **3.00×10^−20^** |
|  |  | 0.1 | 0.066 | 0.0068 | 0.0012 | **9.82×10^−23^** |
|  |  | 0.5 | 0.069 | 0.0069 | 0.0013 | **4.84×10^−24^** |
|  |  | 1 | 0.070 | 0.0069 | 0.0013 | **2.95×10^−24^** |
|  | Longevity | 0.01 | NA | NA | NA | NA |
|  |  | 0.05 | NA | NA | NA | NA |
|  |  | 0.1 | NA | NA | NA | NA |
|  |  | 0.5 | NA | NA | NA | NA |
|  |  | 1 | NA | NA | NA | NA |
|  | Forced Expiratory Volume in 1s (FEV_1_) | 0.01 | NA | NA | NA | NA |
|  |  | 0.05 | NA | NA | NA | NA |
|  |  | 0.1 | NA | NA | NA | NA |
|  |  | 0.5 | NA | NA | NA | NA |
|  |  | 1 | NA | NA | NA | NA |
| **Life-course Cognitive Traits and Proxies** | Childhood Cognitive Ability | 0.01 | 0.062 | 0.0066 | 0.0011 | **5.29×10^−2^1** |
|  |  | 0.05 | 0.095 | 0.0066 | 0.0026 | **1.67×10^−46^** |
|  |  | 0.1 | 0.106 | 0.0066 | 0.0032 | **4.17×10^−58^** |
|  |  | 0.5 | 0.120 | 0.0066 | 0.0041 | **9.28×10^−74^** |
|  |  | 1 | 0.122 | 0.0066 | 0.0042 | **3.92×10^−75^** |
|  | College degree | 0.01 | 0.205 | 0.0067 | 0.0118 | **1.47×10^−206^** |
|  |  | 0.05 | 0.251 | 0.0067 | 0.0175 | **1.60×10^−303^** |
|  |  | 0.1 | 0.265 | 0.0068 | 0.0194 | **0** |
|  |  | 0.5 | 0.280 | 0.0068 | 0.0216 | **0** |
|  |  | 1 | 0.279 | 0.0068 | 0.0214 | **0** |
|  | Years of Education | 0.01 | 0.210 | 0.0067 | 0.0124 | **1.56×10^−216^** |
|  |  | 0.05 | 0.244 | 0.0067 | 0.0167 | **5.61×10^−289^** |
|  |  | 0.1 | 0.265 | 0.0068 | 0.0195 | **0** |
|  |  | 0.5 | 0.283 | 0.0068 | 0.0220 | **0** |
|  |  | 1 | 0.285 | 0.0068 | 0.0223 | **0** |

**Supplementary Table 5**

Multivariate models predicting cognitive (and educational) phenotypes, including all polygenic profile scores together with covariates (age, sex, assessment centre, genotyping batch and array, and ten genetic principal components for population structure; covariate values not shown here). All phenotypes scored such that higher scores indicate better performance. Adjusted *R*^2^ values refer to the polygenic profile scores only (excluding variance explained by the covariates). Statistically significant p-values (after False Discovery Rate correction across 94 tests; threshold: p <0**.**025) shown in bold.

| **Trait Category** | **Trait** | **Verbal-numerical**  **Reasoning**  **(Adj. *R*^2^ = 0.0226)** | | **Memory**  **(Adj. *R*^2^ = 0.0017)** | | **Reaction Time**  **(Adj. *R*^2^ = 0.0012)** | | **Educational**  **Attainment**  **(Adj. *R*^2^ = 0.0333)** | |
| --- | --- | --- | --- | --- | --- | --- | --- | --- | --- |
|  |  | β | p | β | p | β | p | β | p |
| **Vascular-metabolic**  **Diseases** | Coronary Artery Disease | **−0.012** | **0.021** | −0.002 | 0.506 | 0.006 | 0.032 | **−0.034** | **<0.001** |
|  | Stroke: Ischaemic | −0.009 | 0.102 | −0.002 | 0.631 | −0.006 | 0.034 | 0.002 | 0.750 |
|  | Stroke: Cardioembolic | −0.006 | 0.204 | 0.004 | 0.205 | 0.005 | 0.092 | 0.001 | 0.884 |
|  | Stroke: Large Vessel Disease | −0.008 | 0.141 | **−0.010** | **0.002** | 0.002 | 0.410 | **−0.022** | **0.002** |
|  | Stroke: Small Vessel Disease | −0.008 | 0.113 | −0.005 | 0.097 | −0.003 | 0.338 | **−0.028** | **<0.001** |
|  | Type 2 Diabetes | 0.004 | 0.499 | 0.002 | 0.510 | −0.002 | 0.450 | **−**0.005 | 0.484 |
| **Neuro-psychiatric**  **Disorders** | ADHD | −0.007 | 0.170 | −0.003 | 0.253 | −0.004 | 0.194 | **−0.018** | **0.007** |
|  | Alzheimer’s Disease | **−0.018** | **<0.001** | **−0.008** | **0.007** | −0.005 | 0.089 | **−0.034** | **<0.001** |
|  | Autism | **0.018** | **<0.001** | 0.004 | 0.224 | 0.004 | 0.147 | **0.060** | **<0.001** |
|  | Bipolar Disorder | 0.006 | 0.232 | −0.006 | 0.068 | −0.003 | 0.222 | **0.045** | **<0.001** |
|  | Major Depressive Disorder | −0.010 | 0.061 | −0.006 | 0.053 | −0.006 | 0.025 | **−**0.010 | 0.147 |
|  | Schizophrenia | **−0.061** | **<0.001** | **−0.029** | **<0.001** | **−0.028** | **<0.001** | **0.016** | **0.022** |
| **Brain Measures** | Hippocampal Volume | 0.005 | 0.289 | −0.004 | 0.126 | 0.004 | 0.121 | 0.009 | 0.168 |
|  | Intracranial Volume | 0.009 | 0.080 | 0.005 | 0.103 | 0.003 | 0.282 | **0.025** | **<0.001** |
|  | Infant Head Circumference | **0.015** | **0.004** | 0.001 | 0.653 | **−0.010** | **<0.001** | **0.041** | **<0.001** |
| **Physical and**  **Physiological Measures** | BMI | **−0.014** | **0.007** | **0.015** | **<0.001** | 0.003 | 0.311 | **−0.058** | **<0.001** |
|  | Diastolic Blood Pressure | −0.007 | 0.273 | 0.004 | 0.278 | 0.001 | 0.635 | 0.001 | 0.940 |
|  | Height | 0.004 | 0.484 | −0.001 | 0.681 | 0.001 | 0.830 | **0.037** | **<0.001** |
|  | Longevity | 0.003 | 0.526 | 0.005 | 0.068 | 0.002 | 0.491 | - | - |
|  | Forced Expiratory Volume in 1s | 0.009 | 0.076 | −0.004 | 0.143 | −0.003 | 0.222 | - | - |
|  | Systolic Blood Pressure | 0.004 | 0.538 | 0.0001 | 0.974 | −0.005 | 0.212 | 0.015 | 0.082 |
| **Life-course Cognitive Traits**  **and Proxies** | Childhood Cognitive Ability | **0.063** | **<0.001** | **0.011** | **<0.001** | **0.008** | **0.004** | **0.095** | **<0.001** |
|  | College Degree | **0.046** | **<0.001** | 0.004 | 0.285 | 0.005 | 0.193 | **0.140** | **<0.001** |
|  | Years of Education | **0.064** | **<0.001** | −0.003 | 0.360 | 0.005 | 0.187 | **0.162** | **<0.001** |
